# Construction and Validation of a Prediction Model for Killip Classes II–IV During Hospitalisation in Patients With Acute ST-segment Elevation Myocardial Infarction

**DOI:** 10.31083/RCM38402

**Published:** 2025-09-25

**Authors:** Ying Zhou, Guiying Du, Yunqiang Zhang, Mu Guo, Fei Dong, Yufei Zhao, Rui Jing, Yu Song

**Affiliations:** ^1^Department of Heart Failure, TEDA International Cardiovascular Hospital, Tianjin University, 300457 Tianjin, China; ^2^Department of Cardiology, TEDA International Cardiovascular Hospital, Tianjin University, 300457 Tianjin, China; ^3^Department of CCU, TEDA International Cardiovascular Hospital, Tianjin University, 300457 Tianjin, China; ^4^Department of Cardiology, Shanghai Fourth People's Hospital, 200434 Shanghai, China

**Keywords:** ST-segment elevation acute myocardial infarction, primary percutaneous coronary intervention, soluble growth stimulator gene 2 protein, Killip class

## Abstract

**Background:**

To perform a comprehensive assessment of the predictive value of soluble growth stimulator gene 2 protein (sST2) in predicting in-hospital Killip classes II–IV among patients with acute ST-segment elevation myocardial infarction (STEMI). This study aimed to provide more precise prognostic insights for informed clinical decision-making.

**Methods:**

A retrospective cohort study was performed. The clinical records of STEMI patients admitted to Tianjin TEDA International Cardiovascular Hospital and who received primary percutaneous coronary intervention (PPCI) within 24 hours of symptom onset from July 2021 to March 2023 were analyzed. Statistical methodologies, including univariate and multivariate analyses, were applied to identify potential risk factors associated with the development of in-hospital Killip classes II–IV and to construct a reliable prediction model.

**Results:**

Among a total of 232 enrolled STEMI patients, 50 experienced Killip classes II–IV during their hospitalisation. Compared to those with Killip class I, the Killip class II-IV patients presented with significantly elevated sST2 concentrations and a higher heart rate (HR) at the first visit. In contrast, the left ventricular ejection fraction (LVEF) and estimated glomerular filtration rate (eGFR) values in these patients were significantly lower. Multivariate logistic regression analysis revealed that an sST2 level >77.3 ng/mL (odds ratio (OR) = 2.813, 95% confidence interval (CI): 1.201–6.586, *p* = 0.017), a first-visit HR >94 bpm (OR = 7.286, 95% CI: 2.778–19.106, *p* < 0.001), an LVEF <50% (OR = 3.336, 95% CI: 1.458–7.631, *p* = 0.004), and an eGFR <84 mL/(min·1.73 m^2^) (OR = 3.807, 95% CI: 1.556–9.316, *p* = 0.003) were independent risk factors for the occurrence of in-hospital Killip classes II–IV in STEMI patients treated with PPCI. Receiver operating characteristic (ROC) curve analysis, along with decision curve analysis (DCA), indicated that the combined predictive model integrating sST2, first-visit HR, LVEF, and eGFR exhibited a significantly stronger predictive ability compared to any single parameter.

**Conclusion:**

In STEMI patients undergoing PPCI, the combination of sST2, first-visit HR, LVEF, and eGFR can effectively predict patients with Killip classes II–IV during hospitalisation, which may contribute to early intervention and improved patient outcomes.

## 1. Introduction

Currently, cardiovascular disease ranks as the leading cause of mortality globally. Acute ST-segment elevation myocardial infarction (STEMI) remains one of the most serious acute manifestations of coronary artery disease [1]. Over the past few decades, significant advancements have been made in the management of STEMI, particularly with the widespread accessibility of primary percutaneous coronary intervention (PPCI). However, the in-hospital mortality rate for STEMI patients still ranges between 5% and 8%, and the 1-year mortality rate can be as high as 14.3% [2]. Following myocardial infarction, cardiomyocytes experience impaired energy metabolism. Concurrently, inflammatory responses, oxidative stress, ischemia-reperfusion injury, myocardial hypertrophy, and fibrosis occur. These factors collectively result in abnormal myocardial remodelling and facilitate the progression of heart failure (HF) [3]. The typical clinical manifestations include dyspnea, pulmonary rales, peripheral edema, and elevated B-type natriuretic peptide (BNP) levels. Therefore, the early identification of high-risk features of HF is crucial for improving the prognosis of STEMI patients. Growth stimulator gene 2 (ST2) belongs to the interleukin (IL)-1 receptor family and has two subtypes: transmembrane (ST2L) and soluble (sST2). ST2L binds to IL-33 and has cardioprotective effects. These effects mainly include anti-myocardial fibrosis, inhibition of cardiac hypertrophy, reduction of apoptosis, and improvement of cardiac function. During HF, the secretion of sST2 increases. It then competitively binds to IL-33, thereby reducing the cardioprotective effects of the ST2L-IL-33 complex. Both domestic and international HF guidelines have suggested [4, 5] that sST2, an indicator of myocardial fibrosis, is valuable for the risk stratification and prognostic assessment of HF patients. However, there is a paucity of information regarding the use of sST2 to predict the development of Killip class II-IV during hospitalisation in STEMI patients treated with PPCI. Therefore, the objective of this study was to investigate the predictive efficacy of sST2 for the development of Killip class II-IV during the hospitalisation of STEMI patients.

## 2. Information and Methodology

### 2.1 Objectives 

This study retrospectively evaluated a total of 232 patients who presented to the Tianjin TEDA International Cardiovascular Hospital within 24 h of symptom onset between July 2021 and March 2023. These patients were diagnosed with STEMI and received PPCI. The diagnostic criteria for STEMI were derived from the 2017 guidelines for the diagnosis and treatment of STEMI by the European Society of Cardiology (ESC) [6].

**Fig. 1.**
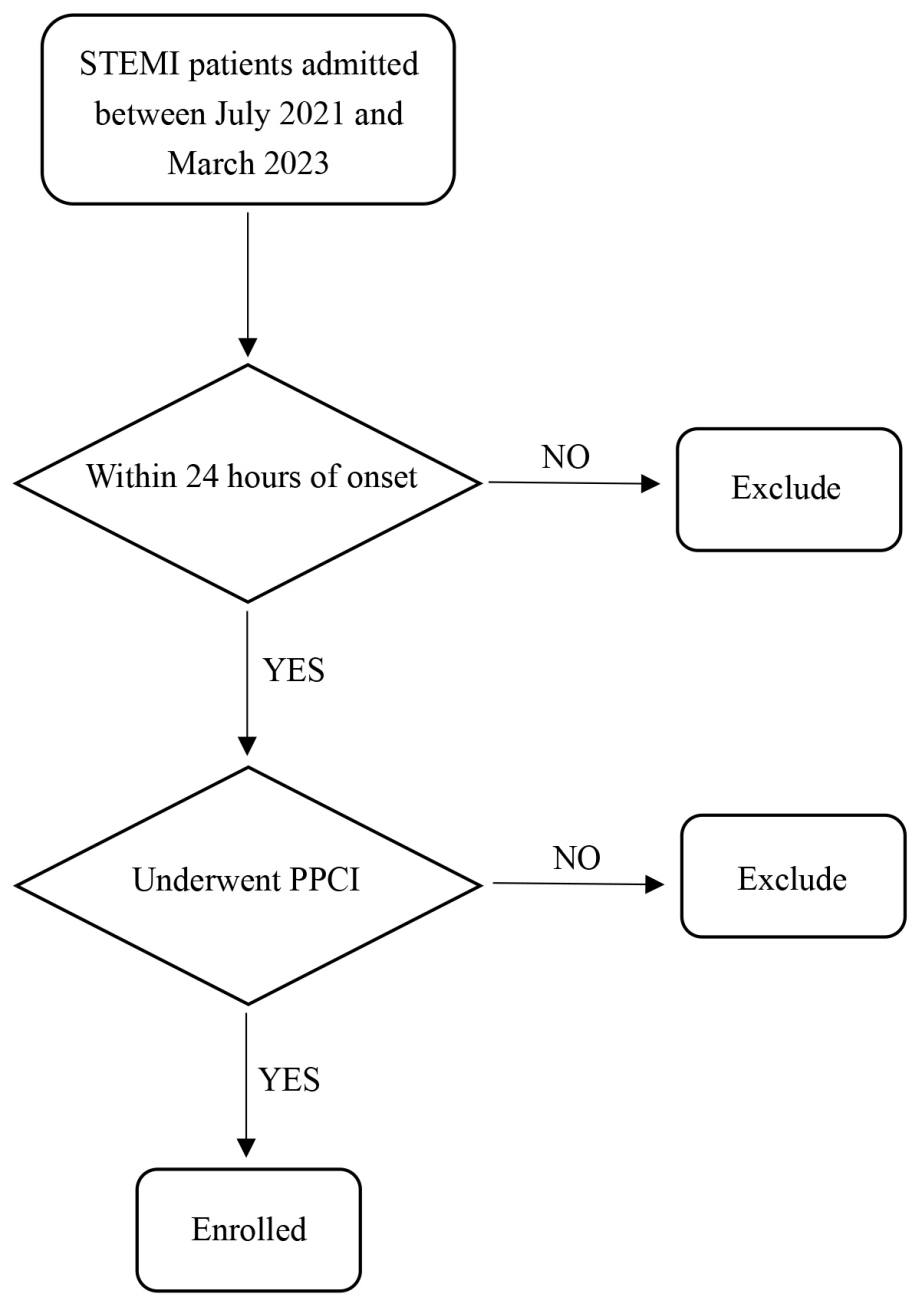
**Flow diagram of patient enrollment**. STEMI, ST-segment elevation myocardial infarction; PPCI, primary percutaneous coronary intervention.

Upon arrival at the emergency department of Chest Pain Centre, once the diagnosis of STEMI was confirmed, the catheterization laboratory was promptly activated, and emergency coronary angiography (CAG) was promptly carried out. The criteria for intraoperative PCI were based on the 2021 Guidelines for Coronary Revascularisation jointly published by the American College of Cardiology (ACC), American Heart Association (AHA), and Society of Cardiovascular Angiography and Interventions (SCAI) [7]. Both pre-operative and post-operative treatments were standardized in accordance with the ESC 2017 guidelines for the diagnosis and treatment of STEMI [6]. A flowchart of patient enrollment is presented in Fig. 1.

The study was approved by the ethics committee of the Tianjin TEDA International Cardiovascular Hospital (ethical approval number: [2023]-0310-1).

### 2.2 Data Collection 

General information: This included gender, age, and body mass index (BMI).

Medical history: Information regarding the onset time of the disease, history of hypertension, diabetes mellitus, smoking status, and alcohol consumption was collected.

Initial vital signs: Temperature, heart rate (HR), respiratory rate (RR), blood pressure (BP), and oxygen saturation were recorded.

Intraoperative data: Door-to-wire time (D-to-W time), infarct-related artery (IRA), IRA pre-PCI thrombolysis in myocardial infarction (TIMI) flow grade, IRA post-PCI TIMI flow grade and the number of stents implanted were collected.

Laboratory tests: Cardiac biomarkers, including high-sensitivity troponin I (cTNI), myoglobin (MYO), and creatine kinase-MB isoenzyme (CK-MB), arterial blood gas analysis, full blood count, renal function tests, liver function tests, electrolyte assays, lipid profiles, random blood glucose (RBG) measurement, C-reactive protein (CRP) determination, sST2 quantification, N-terminal B-type natriuretic peptide precursor (NT-proBNP) assessment, thyroid function tests, bedside cardiac ultrasound examinations, and holter monitor were conducted. All of the above tests (excluding the holter monitor) were completed within 24 hours of admission, while the ambulatory electrocardiogram was completed within 48 hours of admission. The estimated glomerular filtration rate (eGFR) was calculated using the modified simplified modification of diet in renal disease (MDRD) formula.

All sST2 blood samples collected at 12 hours post-PPCI. The study protocol specified that 5 mL of venous blood was collected from each patient using serum separation gel tubes containing clot activator. Following collection, samples were centrifuged at 2095 ×g for 5 minutes at room temperature (18–25 °C) to separate serum, which was then aliquoted and immediately stored at –80 °C until analysis. Serum sST2 concentrations were quantitatively measured using the Leadman CI2000S fully automated chemiluminescence immunoassay analyzer (Beijing, China), with the normal reference range established as 0–35 ng/mL.

### 2.3 Observation Index

In accordance with the 2017 guideline for the diagnosis and treatment of STEMI by the ESC [6], the Killip cardiac function classification was applied to STEMI patients. Patients were categorized into two groups based on Killip classification: class I vs. class II–IV. By comparing the clinical parameters of the two groups of patients, independent predictors of in-hospital Killip class II–IV in STEMI patients were screened out. Subsequently, a prediction model was established and evaluated.

### 2.4 Statistical Analysis 

Normality was evaluated by Kolmogorov-Smirnov test (α = 0.05, *p* ≥ 0.05), showing no significant deviation from normal distribution. Measurement data conforming to a normal distribution were presented as mean ± standard deviation, while those not conforming to a normal distribution were expressed as the median and quartiles (P25, P75). Count data were presented as percentages (%). For continuous variables, intergroup comparisons were performed using the independent samples *t*-test (for two groups) when normally distributed, while the Mann-Whitney U test was employed for non-normally distributed data. Categorical variables were compared using the χ^2^ test or rank-sum test.

The optimal cut-off values of the variables were calculated using the receiver operating characteristic curve (ROC curve), with Youden’s index (Youden’s index = sensitivity + specificity-1) serving as the criterion. Univariate logistic regression and least absolute shrinkage and selection operator (LASSO) regression were utilized to screen for risk factors of heart failure in STEMI patients treated with PPCI. Covariance analysis was performed on the univariate analysis of variance indices, and the screened risk factors were incorporated into multivariate logistic regression to establish a prediction model.

The efficacy of the prediction model was evaluated using ROC curve analysis and decision curve analysis (DCA). Statistical analyses were performed using SPSS 26 (IBM Corporation, Armonk, NY, USA) and R 4.1 (R Foundation for Statistical Computing, Vienna, Austria; glmnet, rmda packages). A *p*-value < 0.05 was considered statistically significant.

## 3. Results

### 3.1 Basic Data 

Among the 232 patients, 190 (81.9%) were male and 42 (18.1%) were female, with a mean age of 59.6 ± 12.0 years. During hospitalisation, 50 patients developed Killip class II–IV. Specifically, 34 of them were classified as Killip class II, 3 as Killip class III, and 13 as Killip class IV. Compared with patients Killip class I, those Killip class II–IV had significantly higher values in terms of age, first-visit HR, NT-proBNP, MYO, CK-MB, alanine aminotransferase (ALT), aspartate aminotransferase (AST), white blood cell count (WBC), neutrophil percentage (N%), CRP, RBG, serum potassium (K^+^), total protein (TP), globulin (GLO), sST2, lactate (LAC), mean HR (holter), and max HR (holter) (*p* < 0.05). Conversely, their left ventricular ejection fraction (LVEF), eGFR, serum chloride (Cl^–^), and max RR (holter) were significantly lower (*p* < 0.05). In the Killip class II-IV group, the proportion of IRA being the left main coronary artery (LM) and left anterior descending branch (LAD) was higher, the proportion of post-PCI TIMI flow grade <3 was significantly higher (*p* < 0.05). However, there was no statistically significant difference in gender, BMI, smoking history, hypertension history, onset time, D-to-W time, IRA pre-PCI TIMI flow grade, number of stents implanted, and lipid profiles. For detailed data, refer to Table 1.

**Table 1.** **Comparison of baseline characteristics of patients**.

Variable		Total (n = 232)	Killip class I (n = 182)	Killip class II–IV (n = 50)	*p*
Age (years)		59.6 ± 12.0	58.7 ± 12.1	62.6 ± 11.2	0.041
Women (%)		42 (18.1)	33 (18.1)	9 (18.0)	0.983
BMI (Kg/m^2^)		24.9 (23.3, 27.0)	25.2 (23.5, 27.1)	24.4 (22.5, 25.9)	0.096
Current smoking (%)		114 (49.1)	92 (50.5)	22 (44.0)	0.412
Drinking history (%)		48 (20.7)	33 (18.1)	15 (30.0)	0.067
Hypertension (%)		130 (56.0)	102 (56.0)	28 (56.0)	0.996
Diabetes (%)		54 (23.3)	38 (20.9)	16 (32.0)	0.099
Onset time (h)		3.0 (2.0, 5.0)	3.0 (2.0, 5.0)	3.3 (2.0, 5.8)	0.538
First vital signs					
	HR (times/minute)	73 (62, 86)	71 (61, 80)	87 (67, 102)	<0.001
	RR (times/minute)	18 (17, 20)	18 (18, 20)	20 (17, 20)	0.241
	SBP (mmHg)	141 (123, 157)	142 (124, 158)	136 (118, 153)	0.125
	DBP (mmHg)	86 (76, 98)	85 (76, 98)	86 (76, 97)	0.963
	MAP (mmHg)	104 (94, 116)	105 (94, 117)	102 (89, 115)	0.566
	SpO_2_ (%)	98 (97, 99)	98 (97, 99)	98 (97, 99)	0.073
	D-to-W time (min)	56 (79, 75)	57 (94, 76)	55 (48, 68)	0.221
IRA					
	LM (%)	4 (1.7)	0 (0.0)	4 (8.0)	0.001
	LAD (%)	120 (51.7)	87 (47.8)	33 (66.0)	
	LCX (%)	18 (7.8)	17 (9.3)	1 (2.0)	
	RCA (%)	89 (38.4)	77 (42.3)	12 (24)	
	Intermediate branch (%)	1 (0.4)	1 (0.5)	0 (0)	
IRA pre-PCI TIMI flow grade					
	Class 0 (%)	41 (17.7)	34 (18.7)	7 (14.0)	0.636
	Class 1 (%)	14 (6.0)	12 (6.6)	2 (4.0)	
	Class 2 (%)	25 (10.8)	18 (9.9)	7 (14.0)	
	Class 3 (%)	152 (65.5)	118 (64.8)	34 (68.0)	
IRA Pos-PCI TIMI flow grade					
	Class 2 (%)	7 (3.0)	2 (1.1)	5 (10.0)	0.005
	Class 3 (%)	225 (97.0)	180 (98.9)	45 (90.0)	
Number of stents implanted					
	1 (%)	193 (83.2)	154 (84.6)	39 (78.0)	0.256
	2 (%)	37 (15.9)	27 (14.8)	10 (20.0)	
	≥3 (%)	2 (0.9)	1 (0.5)	1 (2.0)	
LVEF (%)		53 ± 8	55 ± 7	48 ± 8	<0.001
LV-Dds (mm)		46 (44, 48)	46 (44, 48)	46 (43, 49)	0.616
NT-proBNP (pg/mL)		1088 (368, 1815)	940 (314, 1483)	1570 (817, 2971)	<0.001
cTnI (pg/mL)		25.6 (21.4, 26.7)	25.6 (16.7, 26.7)	25.6 (25.4, 26.6)	0.112
MYO (ng/mL)		104.5 (53.0, 297.6)	93.5 (48.5, 217.7)	251.2 (90.2, 539.6)	<0.001
CK-MB (ng/mL)		146.7 (68.5, 288.0)	136.7 (57.2, 262.4)	206.5 (113.0, 288.0)	0.003
WBC (10^9^/L)		9.9 (8.4, 12.3)	9.8 (8.3, 12.2)	11.1 (9.0, 14.1)	0.023
N%		74.1 ± 8.0	73.2 ± 7.8	77.1 ± 7.7	0.002
RBC (10^12^/L)		4.5 ± 0.6	4.5 ± 0.6	4.5 ± 0.6	0.511
HB (g/L)		138 ± 17	138 ± 17	139 ± 19	0.559
HCT (%)		41.3 ± 4.8	41.0 ± 4.8	41.7 ± 5.6	0.415
PLT (10^9^/L)		213 (180, 253)	218 (180, 255)	206 (178, 247)	0.319
CRP (mg/L)		4.9 (2.4, 13.3)	4.9 (2.2, 9.6)	12.3 (3.3, 35.2)	<0.001
ALP (U/L)		78.0 (65.4, 91.0)	77.7 (65.9, 91.3)	84.1 (65.0, 90.0)	0.569
ALT (U/L)		53 (30, 74)	45 (29, 69)	73 (55, 113)	<0.001
AST (U/L)		196 (100, 315)	164 (85, 259)	340 (205, 464)	<0.001
RBG (mmol/L)		8.5 (7.0, 10.7)	8.3 (6.9, 10.4)	9.2 (7.3, 12.6)	0.007
Cr (μmol/L)		69 (59, 79)	67 (58, 76)	75 (65, 100)	0.001
BUN (mmol/L)		6.0 (5.0, 7.0)	5.8 (4.9, 7.0)	7.7 (5.4, 9.7)	<0.001
UA (μmol/L)		346 (285, 402)	345 (284, 388)	363 (292, 441)	0.097
eGFR [mL/(min⋅1.73 m^2^)]		106.7 (82.3, 129.3)	108.1 (89.5, 130.9)	86.8 (57.4, 119.9)	0.004
K^+^ (mmol/L)		3.9 (3.7, 4.2)	3.9 (3.7, 4.2)	4.1 (3.8, 4.6)	0.005
NA^+^ (mmol/L)		139 (138, 141)	139 (138, 141)	139 (137, 141)	0.081
CL^–^ (mmol/L)		105 (105, 107)	106 (103, 108)	103 (102, 106)	0.001
TP (g/L)		65 (63, 68)	65 (62, 67)	66 (64, 71)	0.011
ALB (g/L)		39 (38, 41)	40 (38, 41)	39 (37, 41)	0.159
GLO (g/L)		26 (24, 28)	25 (23, 27)	28 (26, 31)	<0.001
TCHOL (mmol/L)		4.5 (4.0, 5.1)	4.5 (4.0, 5.1)	4.6 (4.0, 5.3)	0.658
TG (mmol/L)		1.5 (1.0, 2.2)	1.6 (1.0, 2.4)	1.3 (0.9, 2.0)	0.193
LDL-C (mmol/L)		2.9 (2.4, 3.4)	2.87 (2.4, 3.4)	3.0 (2.4, 3.6)	0.705
HDL-C (mmol/L)		1.0 (0.8, 1.2)	1.0 (0.8, 1.1)	1.0 (0.9, 1.2)	0.059
sST2 (ng/mL)		47.8 (27.2, 92.6)	40.0 (25.1, 77.6)	90.1 (44.3, 185.6)	<0.001
TSH (mIU/L)		1.1 (0.7, 1.9)	1.1 (0.7, 1.9)	1.2 (0.7, 1.9)	0.583
LAC (mmol/L)		1.8 (1.3, 2.5)	1.7 (1.2, 2.3)	2.1 (1.5, 3.2)	0.006
Holter monitor					
	Mean HR (bpm)	71 (66, 82)	69 (64, 78)	82 (72, 89)	<0.001
	Max HR (bpm)	106 (97, 115)	104 (95, 114)	113 (103, 121)	0.001
	Total ventricular rhythms (times)	53 (5, 1226)	37 (4, 724)	180 (8, 2026)	0.119
	Total atrial rhythms (times)	48 (9, 335)	44 (9, 320)	57 (9, 640)	0.300
	Max RR (s)	1.3 (1.2, 1.5)	1.4 (1.2, 1.5)	1.2 (1.0, 1.4)	0.002

Note: BMI, body mass index; HR, heart rate; RR, respiratory rate; SBP, systolic blood pressure; DBP, diastolic blood pressure; MAP, mean arterial pressure; SpO_2_, oxygen saturation; IRA, infarct-related artery; LM, left main coronary artery; LAD, left anterior descending branch; LCX, left circumflex branch; RCA, right coronary artery; LVEF, left ventricular ejection fraction; LV-Dds, left ventricular end-diastolic inner diameter; NT-proBNP, N-terminal B-type natriuretic peptide precursor; cTnI, high-sensitivity troponin I; MYO, myoglobin; CK-MB, creatine kinase-MB isoenzyme; WBC, white blood cell count; N%, neutrophil percentage; RBC, red blood cell; HB, haemoglobin; HCT, hematocrit; PLT, platelet count; CRP, C-reactive protein; ALP, alkaline phosphatase; ALT, alanine aminotransferase; AST, aspartate aminotransferase; RBG, random blood glucose; Cr, creatinine; BUN, blood urea nitrogen; UA, uric acid; eGFR, estimated glomerular filtration rate; TP, total protein; ALB, albumin; GLO, globulin; TCHOL, total cholesterol; TG, triglycerides; LDL-C, low-density lipoprotein cholesterol; HDL-C, high-density lipoprotein cholesterol; sST2, soluble growth-stimulating expressed gene 2 protein; TSH, thyrotropin; LAC, lactate; D-to-W, door-to-wire; PCI, percutaneous coronary intervention; TIMI, thrombolysis in myocardial infarction.

### 3.2 Calculation of Optimal Cut-off Values Using ROC Curves 

The area under the curve (AUC) of the ROC curves for the first-visit HR, NT-proBNP, eGFR, sST2, and mean HR (holter) were analyzed individually. The Youden’s Index was utilized to determine the optimal cut-off values. The results are presented in Table 2.

**Table 2.** **Optimal cut-off values for ROC curve calculation**.

Variable	AUC	95% CI	Cutoff value	Youden’s index	Specificity	Sensitivity	*p*
First-visit HR (bpm)	0.681	0.587–0.776	94	0.378	91.8%	46.0%	<0.001
NT-proBNP (pg/mL)	0.668	0.580–0.756	1367.05	0.309	70.9%	60.0%	<0.001
eGFR [mL/(min⋅1.73 m^2^)]	0.634	0.540–0.729	84	0.297	79.7%	50.0%	0.004
sST2 (ng/mL)	0.729	0.649–0.809	77.3	0.364	76.4%	60.0%	<0.001
Mean HR (holter) (bpm)	0.726	0.646–0.806	74	0.390	67.0%	72.0%	<0.001

Note: AUC, area under the curve; CI, confidence interval; HR, heart rate; NT-proBNP, N-terminal B-type natriuretic peptide precursor; eGFR, estimated glomerular filtration rate; sST2, soluble growth-stimulating expressed gene 2 protein; ROC, receiver operating characteristic.

### 3.3 Univariate Analysis 

Logistic regression was performed to conduct a univariate analysis of each factor. The results indicated that the factors significantly influencing the occurrence of heart failure were as follows: first-visit HR, IRA, IRA Post-PCI TIMI flow grade, LVEF, NT-proBNP, MYO, CK-MB, WBC, N%, CRP, ALT, AST, RBG, eGFR, sST2, LAC, mean HR (holter), and max HR (holter). The detailed data can be found in Table 3.

**Table 3.** **One-way logistic regression analysis of Killip class II–IV occurring during hospitalisation in STEMI patients**.

Variable	Wald χ^2^	β	OR	SE	95% CI	*p*
Age (per SD years)	3.121	0.263	1.301	0.149	0.972–1.743	0.077
First-visit HR >94 bpm	33.040	2.250	9.484	0.391	4.404–20.423	<0.001
IRA (LM & LAD)	10.207	1.134	3.108	0.355	1.550–6.231	0.001
IRA Post-PCI TIMI flow grade <3	7.285	2.303	10.000	0.853	1.879–53.230	0.007
LVEF <50%	20.090	1.508	4.518	0.336	2.336–8.737	<0.001
NT-proBNP >1367.05 pg/mL	14.673	1.269	3.556	0.331	1.858–6.804	<0.001
MYO (per IQR ng/mL)	7.290	0.134	1.144	0.050	1.038–1.261	0.007
CK-MB (per 100 ng/mL)	7.115	0.526	1.692	0.197	1.150–2.490	0.008
WBC (per 5 × 10^9^/L)	9.945	0.689	1.992	0.218	1.298–3.056	0.002
N% (per SD)	9.363	0.518	1.679	0.169	1.205–2.339	0.002
CRP (per 10 mg/L)	16.175	0.377	1.457	0.094	1.213–1.751	<0.001
ALT (per 40 U/L)	13.931	0.473	1.606	0.127	1.252–2.059	<0.001
AST (per 200 U/L)	23.267	1.072	2.923	0.222	1.890–4.519	<0.001
RBG (per 5 mmol/L)	7.092	0.499	1.647	0.187	1.141–2.379	0.008
eGFR <84 mL/(min⋅1.73 m^2^)	16.375	1.366	3.919	0.338	2.022–7.594	<0.001
sST2 >77.3 ng/mL	21.905	1.579	4.849	0.337	2.503–9.392	<0.001
LAC (per 2 IQR mmol/L)	8.246	0.701	2.015	0.244	1.249–3.250	0.004
Mean HR (holter) >74 bpm	20.184	1.557	4.744	0.347	2.405–9.358	<0.001
Max HR (holter) (per 20 bpm)	10.140	0.634	1.885	0.199	1.276–2.785	0.001
Max RR (holter) (per IQRs)	3.252	–0.319	0.727	0.177	0.514–1.028	0.071

Note: OR, odds ratio; CI, confidence interval; HR, heart rate; IRA, infarct-related artery; LM, left main coronary artery; LAD, left anterior descending branch; LVEF, left ventricular ejection fraction; NT-proBNP, N-terminal B-type natriuretic peptide precursor; MYO, myoglobin; CK-MB, creatine kinase-MB isoenzyme; WBC, white blood cell count; N%, neutrophil percentage; CRP, C-reactive protein; ALT, alanine aminotransferase; AST, aspartate aminotransferase; RBG, random blood glucose; eGFR, estimated glomerular filtration rate; sST2, soluble growth-stimulating expressed gene 2 protein; LAC, lactate; SD, standard deviation; IQR, interquartile spacing; PCI, percutaneous coronary intervention; TIMI, thrombolysis in myocardial infarction; RR, respiratory rate; STEMI, ST-segment elevation myocardial infarction.

### 3.4 LASSO Regression Analysis 

**Fig. 2.**
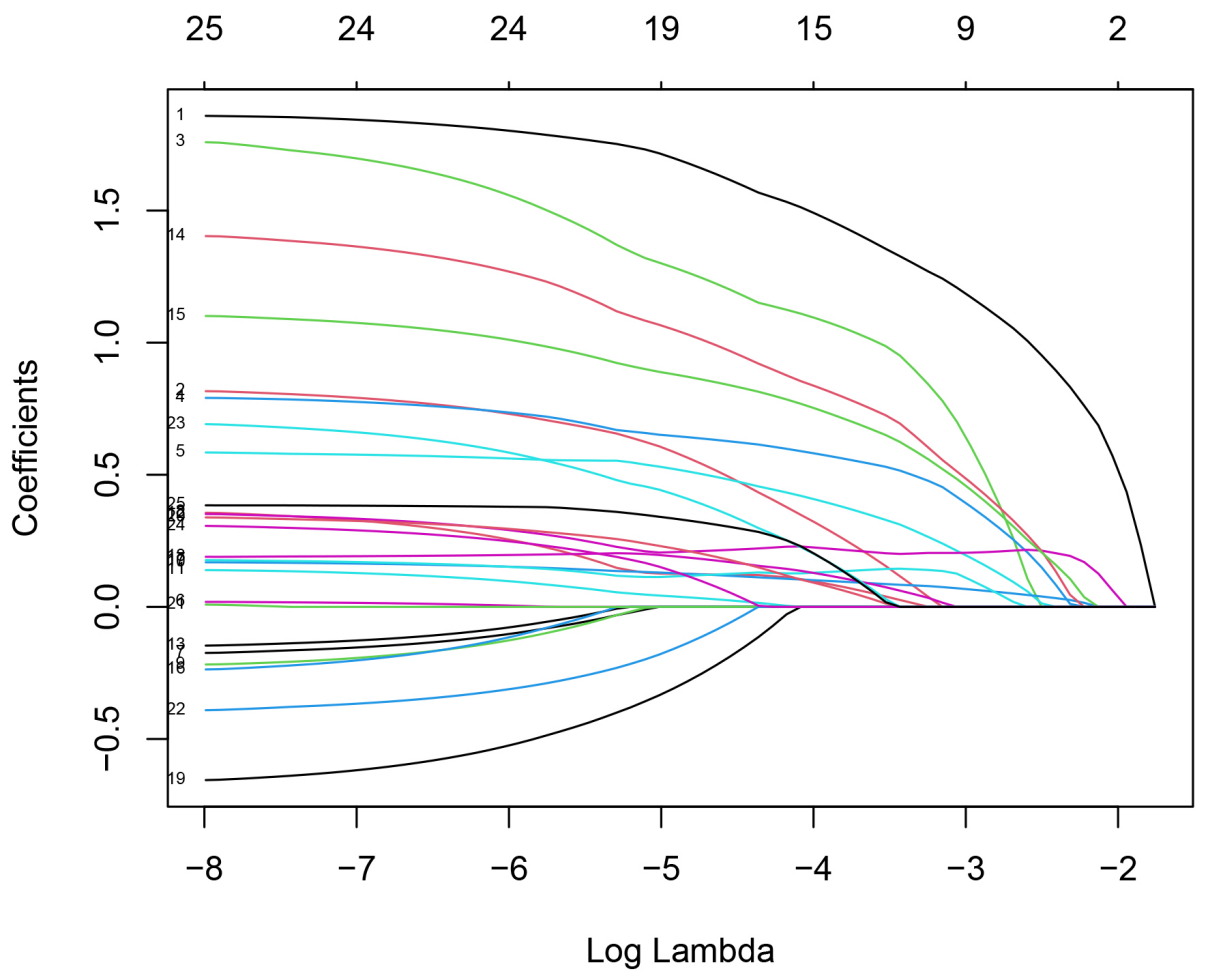
**LASSO regression coefficient relationship**. Note: The horizontal axis below represents log(λ), the vertical axis represents the Lasso regression coefficients, and the horizontal axis above indicates the number of independent variables. LASSO, least absolute shrinkage and selection operator.

**Fig. 3.**
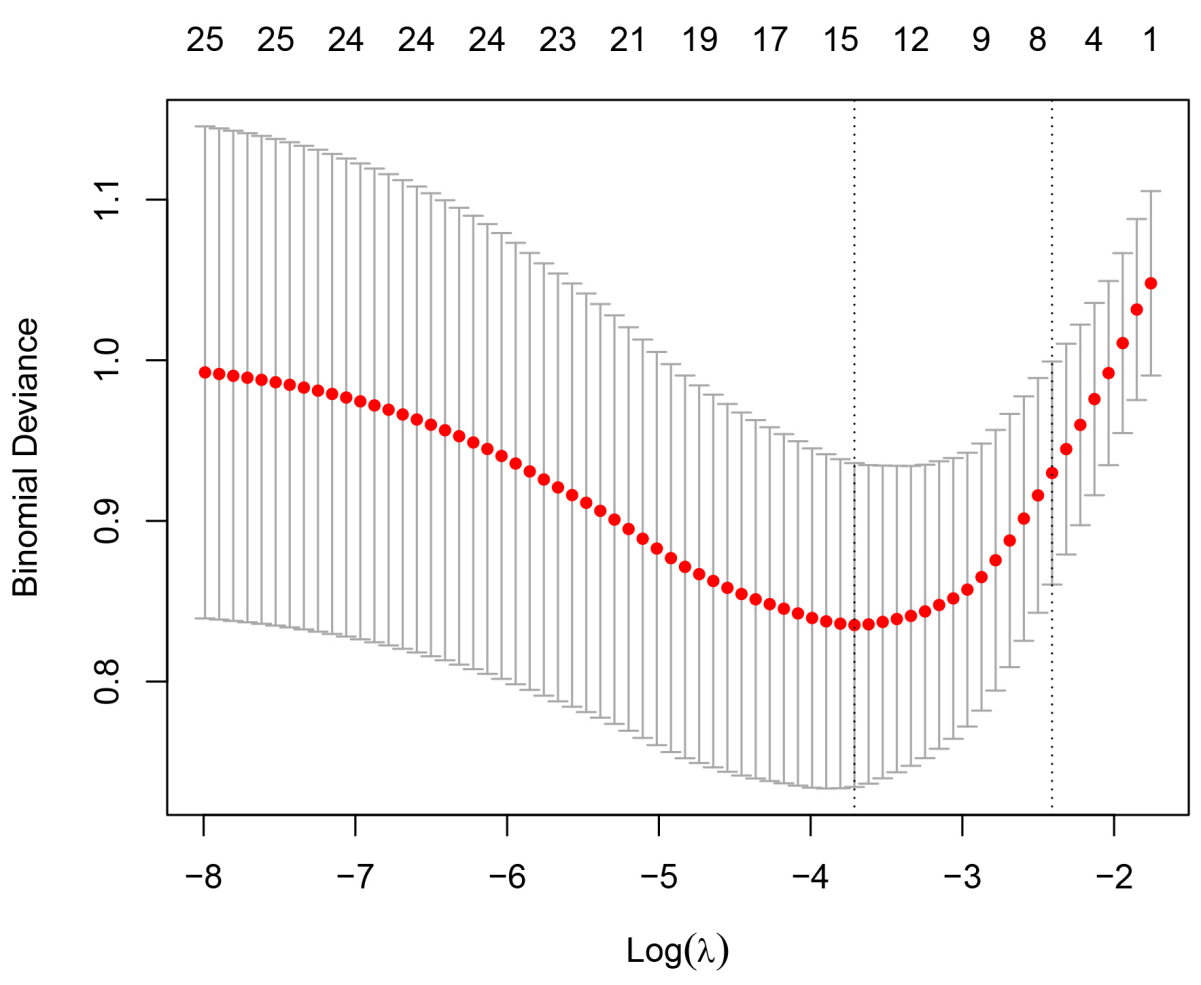
**LASSO regression coefficient relationship**. Note: The dashed vertical lines represent the minimum mean square error and the distance minimum mean square error plus one standard error (optimal solution). The log(λ) value corresponding to the dashed vertical line on the right is the optimal λ value corresponding to the distance minimum mean square error plus one standard error, which is 0.022. LASSO, least absolute shrinkage and selection operator.

Eighteen statistically significant variables mentioned above were incorporated into the LASSO regression analysis. Additionally, age, gender, BMI, history of hypertension, diabetes mellitus, smoking history, and history of alcohol consumption were simultaneously included in the LASSO regression analysis for correction. When the smallest mean squared error occurred at λ = 0.022, the penalty value corresponding to the dotted line on the right-hand side was the lowest. At this point, a total of six variables with non-zero coefficients were screened out. These variables were: first-visit HR >94 bpm, LVEF <50%, CRP (per 10 mg/L), ALT (per 40 U/L), eGFR <84 mL/(min⋅1.73 m^2^), and sST2 >77.3 ng/mL. These were the variables for which the LASSO regression achieved the best fit. For a visual representation, please refer to Figs. 2,3.

### 3.5 Covariance Analysis 

Variables including first-visit HR >94 bpm, LVEF <50%, CRP (per 10 mg/L), ALT (per 40 U/L), eGFR <84 mL/(min⋅1.73 m^2^), and sST2 >77.3 ng/mL were subjected to covariance analysis. The tolerance values (referred to as tolerance or Tol) for all these variables were greater than 0.1, and the variance inflation factor (VIF) for each variable was less than 2. These results indicated the absence of multicollinearity, as presented in Table 4.

**Table 4.** **Covariance analysis of predictors of Killip class II–IV during hospitalisation in patients with STEMI**.

Variable	Tol	VIF
First-visit HR >94 bpm	0.834	1.199
LVEF <50%	0.903	1.107
CRP (per 10 mg/L)	0.833	1.201
ALT (per 40 U/L)	0.792	1.263
eGFR <84 mL/(min⋅1.73 m^2^)	0.879	1.138
sST2 >77.3 ng/mL	0.799	1.251

Note: Tol, tolerance; VIF, variance inflation factor; HR, heart rate; bpm, beats/minute; LVEF, left ventricular ejection fraction; CRP, C-reactive protein; ALT, alanine aminotransferase; eGFR, estimated glomerular filtration rate; sST2, soluble growth-stimulated expressed gene 2 protein; STEMI, ST-segment elevation myocardial infarction.

### 3.6 Multifactorial Analysis 

A multifactorial analysis was conducted by incorporating the above-mentioned six variables into a logistic regression equation. The analysis revealed that an sST2 level >77.3 ng/mL, first-visit HR >94 bpm, LVEF <50%, and eGFR <84 mL/(min⋅1.73 m^2^) were independent risk factors for the Killip class II–IV occurring during hospitalisation in STEMI patients treated with PPCI. The Hosmer-Lemeshow test indicated a good model fit (χ^2^ = 8.770, df = 5, *p* = 0.119). The detailed data can be found in Table 5.

**Table 5.** **Multifactorial logistic regression analysis of Killip class II–IV occurring during hospitalisation in STEMI patients**.

Variable	Wald χ^2^	β	OR	SE	95% CI	*p*
sST2 >77.3 ng/mL	5.678	1.034	2.813	0.434	1.201–6.586	0.017
First-visit HR >94 bpm	16.299	1.986	7.286	0.492	2.778–19.106	<0.001
LVEF <50%	8.140	1.205	3.336	0.422	1.458–7.631	0.004
eGFR <84 mL/(min⋅1.73 m^2^)	8.571	1.337	3.807	0.457	1.556–9.316	0.003

Note: OR, odds ratio; CI, confidence interval; sST2, soluble growth-stimulated expressed gene 2 protein; HR, heart rate; LVEF, left ventricular ejection fraction; eGFR, estimated glomerular filtration rate; STEMI, ST-segment elevation myocardial infarction.

### 3.7 Predictive Efficacy Tested by ROC Curve 

**Fig. 4.**
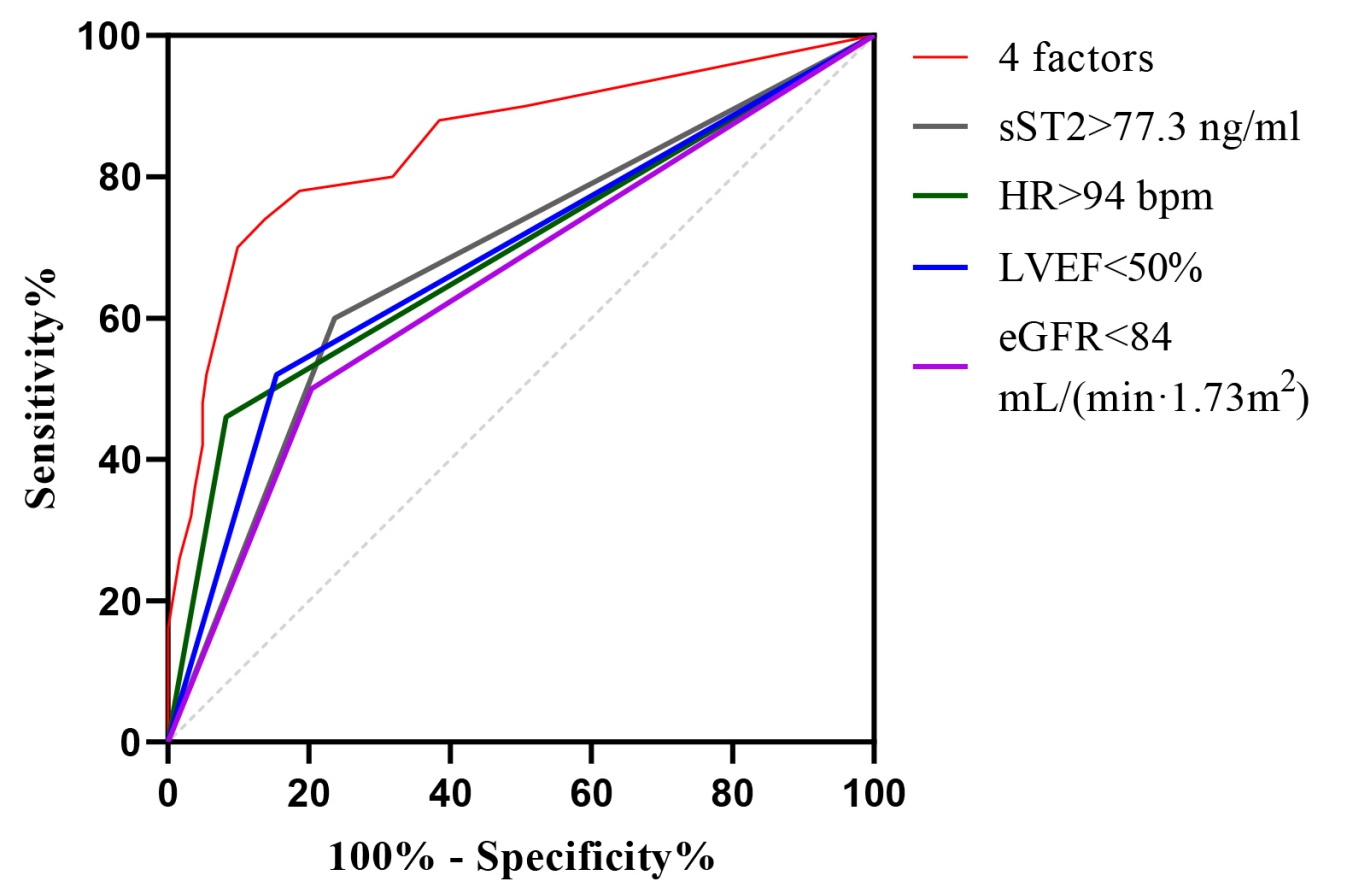
**ROC curve analysis of sST2, HR, LVEF, and eGFR alone and in combination for predictive modeling**. ROC, receiver operating characteristic; sST2, soluble growth-stimulated expressed gene 2 protein; HR, heart rate; LVEF, left ventricular ejection fraction; eGFR, estimated glomerular filtration rate.

When sST2 was combined with the first-visit HR, LVEF, and eGFR to predict the development of Killip class II-IV in STEMI patients treated with PPCI, the AUC was 0.846 (95% CI: 0.778–0.915), with a *p*-value of <0.001. The sensitivity was 70.0% and the specificity was 89.6%. These efficacy metrics were superior to those of any single test. For a visual demonstration, refer to Fig. 4 and Table 6.

**Table 6.** **AUC by ROC analysis**.

Variable	AUC	SE	95% CI	Sensitivity (%)	Specificity (%)	*p*
4 factors	0.846	0.034	0.778–0.915	70.0	89.6	<0.001
sST2 >77.3 ng/mL	0.682	0.045	0.594–0.769	60.0	76.4	<0.001
First-visit HR >94 bpm	0.689	0.047	0.596–0.782	46.0	91.8	<0.001
LVEF <50%	0.683	0.045	0.593–0.774	56.0	78.0	<0.001
eGFR <84 mL/(min⋅1.73 m^2^)	0.648	0.046	0.557–0.739	50.0	79.7	0.001

Note: AUC, area under the curve; ROC, receiver operating characteristic; CI, confidence interval; sST2, soluble growth-stimulated expressed gene 2 protein; LVEF, left ventricular ejection fraction; eGFR, estimated glomerular filtration rate; HR, heart rate.

### 3.8 Predictive Efficacy Tested by by DCA 

**Fig. 5.**
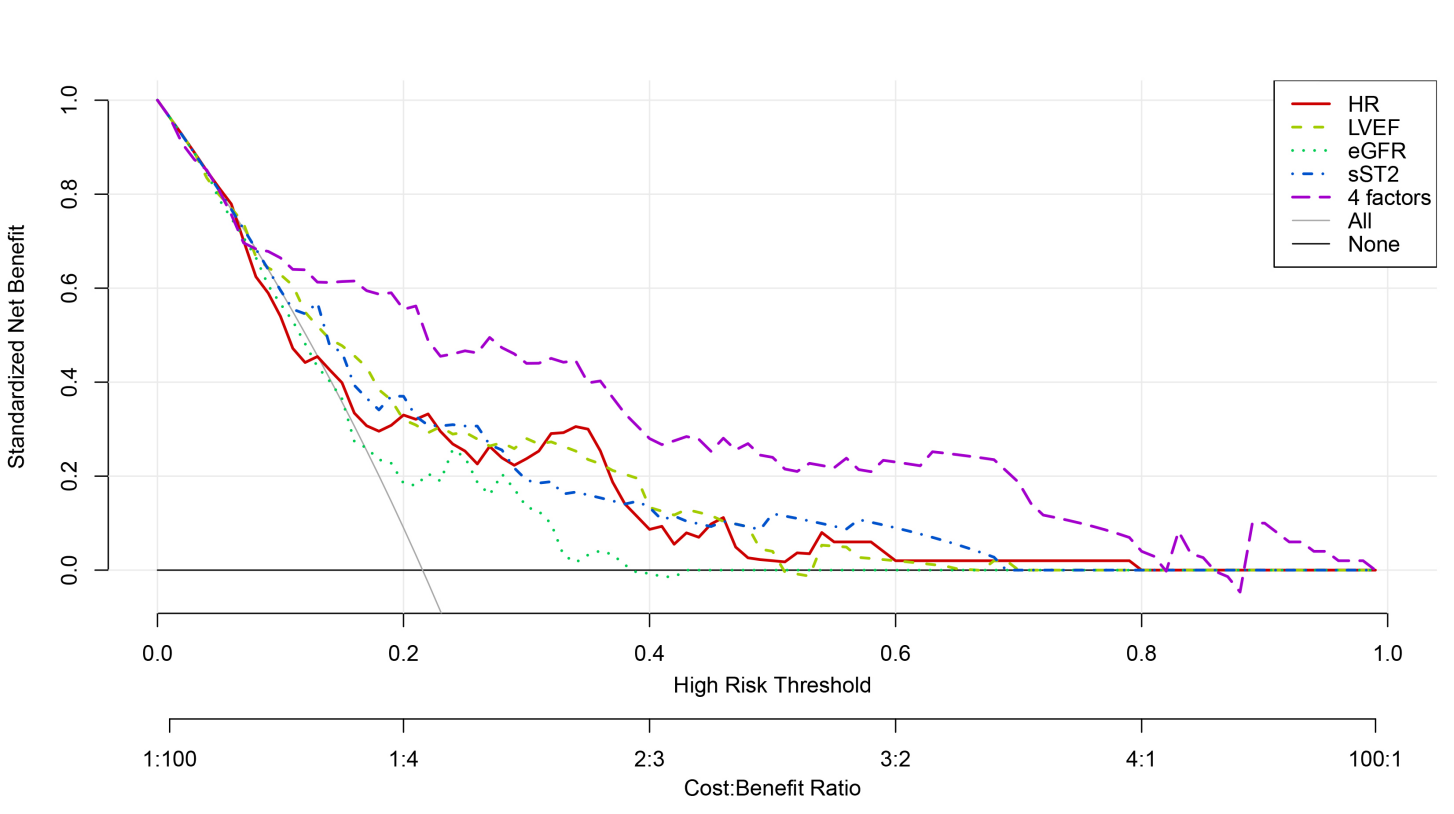
**Decision curves of sST2, HR, LVEF, eGFR alone and in combination to predict Killip class II-IV in-hospital in PPCI-treated STEMI patients**. sST2, soluble growth-stimulated expressed gene 2 protein; HR, heart rate; LVEF, left ventricular ejection fraction; eGFR, estimated glomerular filtration rate; PPCI, primary percutaneous coronary intervention; STEMI, ST-segment elevation myocardial infarction.

The four-marker model (purple dashed line) showed clinical utility when its net benefit exceeded the “all-intervention” (gray solid line) and “no-intervention” (black horizontal line) thresholds, with superior net benefit at threshold probabilities of 0.09–0.81 (Fig. 5). This wide threshold probability range suggests substantial clinical value for the model’s application in practice.

## 4. Discussion

In this study, by analyzing the relationship between clinical indicators and the development of Killip class II–IV during hospitalisation in STEMI patients treated with PPCI, we demonstrated that sST2 was an independent predictor of in-hospital Killip class II–IV development in STEMI patients. Moreover, the predictive efficacy of the combination of sST2, first-visit HR, LVEF, and eGFR was superior to that of any single indicator.

Compared to other biomarkers such as NT-proBNP, the advantage of sST2 lies in its concentration being unaffected by age, renal function, intravascular volume, BMI, or atrial fibrillation [8]. This relative independence from common heart failure comorbidities implies its potential superiority in the prediction of heart failure [8]. NT-proBNP is a traditional and internationally recognized biomarker for heart failure but was not included in the final predictive model of this study. We considered two reasons for the lower-than-expected admission NT-proBNP levels in our study patients, which may have compromised its ability to objectively predict heart failure outcomes. First, the BMI of STEMI patients included in this study was generally high. The median BMI for all patients was 24.87 kg/m^2^, with non-heart failure patients having a median BMI of 25.16 kg/m^2^ and heart failure patients a median BMI of 24.14 kg/m^2^ (no statistically significant difference between the groups). A BMI between 24.0 and 27.9 is classified as overweight. A study has confirmed that NT-proBNP levels are inversely correlated with BMI [9], possibly due to hemodilution, increased degradation of NT-proBNP by adipose tissue, and alterations in ventricular function. Second, acute myocardial infarction (AMI) patients often experience significant diaphoresis due to severe pain before hospitalisation or reduced oral intake, leading to intravascular volume depletion upon admission. Consequently, myocardial mechanical stress may not be significantly increased, resulting in normal or only mildly elevated NT-proBNP levels in the early hospital phase.

The IL-33-ST2L pathway can immunologically inhibit the development of atherosclerosis via helper T-cell 2 and macrophage 2 phenotype responses. Conversely, high levels of sST2 may promote plaque progression [10]. Therefore, sST2 can be regarded as a marker for both early and late post-infarction remodelling. Jenkins *et al*. [11] classified a cohort of 1401 AMI patients into three cardiovascular risk classes according to early sST2 values. Class 2 (37 < sST2 ≤ 72.3 ng/mL) and class 3 (sST2 >72.3 ng/mL) were associated with a higher risk of death within the first 30 days and during the first 5-year follow-up. AMI patients with sST2 >72.3 ng/mL are more prone to the activation of neurohormonal and fibrotic signalling pathways, which increases the risk of adverse myocardial remodelling and heart failure [11]. For AMI patients during hospitalisation, sST2 values can guide discharge decisions, and a 30% reduction in sST2 values at discharge is recommended [12]. In this study, sST2 was measured early (within 24 hours) in STEMI patients treated with PPCI. Based on the ROC curve, the optimal cut-off value for sST2 was 77.3 ng/mL. This was confirmed by one-way logistic regression analysis, LASSO regression analysis, and multifactorial logistic regression analysis, indicating that sST2 >77.3 ng/mL is an independent risk factor for the development of HF in STEMI patients. The 77.3 ng/mL value obtained in this study is close to the 72.3 ng/mL value derived by Jenkins *et al*. [11], further validating the accuracy and reliability of this study.

Acute kidney injury (AKI) and chronic kidney disease (CKD) are indicators of poor prognosis in AMI. As renal function deteriorates, numerous metabolic pathways are disrupted. These include alterations in cardiac volume and pressure status, accelerated atherosclerosis, electrolyte metabolism disorders, the presence of uremic toxins, and oxidative stress, all of which can reduce cardiac function and lead to heart failure [13]. The present study revealed that eGFR levels were significantly lower in the heart failure group compared to the non-heart failure group, and renal insufficiency was an independent risk factor for in-hospital heart failure in STEMI patients. This is consistent with previous studies. Yandrapalli *et al*. [14] found that among 237,549 AMI survivors, 13.8% had concurrent AKI, 16.5% had concurrent CKD, and 3.4% had concurrent end-stage renal disease (ESRD). Moreover, in-hospital heart failure was more prevalent among AMI patients with renal insufficiency. In patients with renal insufficiency, elevated fibroblast growth factor levels are associated with an increased risk of left ventricular hypertrophy, which is related to diastolic dysfunction, HF, and death [15].

It is widely recognized that the admission heart rate is an important predictor of in-hospital mortality in patients with acute coronary syndrome (ACS). Jensen *et al*. [16] proposed that a heart rate >80 bpm in ACS patients should be considered a marker of adverse events during hospitalisation, regardless of the ACS type. In this study, patients in the HF group had a significantly higher initial heart rate than those in the non-HF group. Based on the optimal cut-off value calculated from the ROC curve, a heart rate >94 beats/min was an independent risk factor for in-hospital heart failure in STEMI patients treated with PPCI.

Our study has several limitations. We acknowledge that Killip class II–IV serves as a clinical severity indicator rather than a definitive HF diagnosis, which ideally requires comprehensive assessment incorporating biomarkers (BNP/NT-proBNP) and imaging studies. This may introduce diagnostic misclassification bias. As a single-center study with limited sample size, external validation through multicenter cohorts is needed. Unmeasured confounders (e.g., socioeconomic factors, medication adherence) were not systematically assessed. In future studies, we plan to implement this risk stratification tool in clinical practice to guide personalized treatment decisions and ultimately improve patient outcomes.

## 5. Conclusion

This study verifies that sST2 is an independent predictor for the development of in-hospital Killip class II-IV in STEMI patients. The combination of sST2, first-visit HR, LVEF, and eGFR can enhance the predictive value of Killip class II–IV development in STEMI patients, facilitating early risk stratification and has the potential to improve outcomes in these patients.

## Availability of Data and Materials

The datasets used and analyzed during the current study are available from the corresponding author on reasonable request.
